# Production of Sorbitol via Hydrogenation of Glucose over Ruthenium Coordinated with Amino Styrene-co-maleic Anhydride Polymer Encapsulated on Activated Carbon (Ru/ASMA@AC) Catalyst

**DOI:** 10.3390/molecules28124830

**Published:** 2023-06-17

**Authors:** Xiaorui Yang, Xiaotong Li, Jing Zhao, Jinhua Liang, Jianliang Zhu

**Affiliations:** College of Biotechnology and Pharmaceutical Engineering, Nanjing Tech University, Nanjing 211816, China; yangxiaorui@njtech.edu.cn (X.Y.); lixt0825@163.com (X.L.); zhaojing1112@163.com (J.Z.); jhliang@njtech.edu.cn (J.L.)

**Keywords:** Ru/ASMA@AC catalyst, coordination, glucose conversion, sorbitol yield, confine

## Abstract

Sorbitol, a product primarily derived from glucose hydrogenation, has extensive applications in the pharmaceutical, chemical and other industries. Amino styrene-co-maleic anhydride polymer encapsulated on activated carbon (Ru/ASMA@AC) catalysts were developed for efficient glucose hydrogenation and were prepared and confined Ru by coordination with styrene-co-maleic anhydride polymer (ASMA). Through single-factor experiments, optimal conditions were determined to be 2.5 wt.% ruthenium loading and a catalyst usage of 1.5 g, 20% glucose solution at 130 °C, reaction pressure of 4.0 MPa, and a stirring speed of 600 rpm for 3 h. These conditions achieved a high glucose conversion rate of 99.68% and a sorbitol selectivity of 93.04%. Reaction kinetics testing proved that the hydrogenation of glucose catalyzed by Ru/ASMA@AC was a first-order reaction, with a reaction activation energy of 73.04 kJ/mol. Furthermore, the catalytic performance of the Ru/ASMA@AC and Ru/AC catalysts for glucose hydrogenation were compared and characterized by various detection methods. The Ru/ASMA@AC catalyst exhibited excellent stability after five cycles, whereas the traditional Ru/AC catalyst suffered from a 10% decrease in sorbitol yield after three cycles. These results suggest that the Ru/ASMA@AC catalyst is a more promising candidate for high-concentration glucose hydrogenation due to its high catalytic performance and superior stability.

## 1. Introduction

Sorbitol is a hexabasic alcohol that possesses excellent moisturizing and emulsifying properties and stands out as an important industrial raw material [1,2]. It is odorless, non-toxic, and sweet in taste with microbial-inhibiting properties, making it an excellent food and cosmetics additive [3,4,5,6]). As a platform molecule, by means of different transformation processes, sorbitol can give rise to bulk chemicals ethylene glycol (EG), propylene glycol (PG), glycerol, lactic acid and isosorbide and alkanes, so it serves as a raw material to produce substances that are commonly derived from fossil resources [7,8,9,10,11]. Additionally, sorbitol is an important intermediate for the synthesis of vitamin C and is widely used in the daily chemical industry, pharmacy, papermaking, coating and alternative energy, and other fields [12,13].

Sorbitol has an annual production of over 700,000 tons, and the primary method of production involves glucose catalytic hydrogenation [12,14,15,16]. Glucose can be derived from inexpensive, renewable biomass sources such as starch, cellulose, and straw through hydrolysis [17]. This makes the hydrogenation process environmentally friendly and supports the rational utilization and preservation of non-renewable resources. Additionally, it promotes energy conservation and reduces carbon dioxide emissions [18,19,20,21,22].

The catalyst has always been the core of research on the catalytic hydrogenation of glucose. Conventionally, Raney Nickel has aroused great interest in sorbitol production from glucose hydrogenation owing to its low cost and good catalytic activity [23]. However, the use of Raney nickel or nickel (Ni) catalysts in the industry has apparent shortcomings, such as their flammable nature and side reactions causing less selectivity to sorbitol, along with nickel leaching. Furthermore, severe leaching of Ni also brings large amounts of wastewater contaminated with metal ions [24,25]. As a result, alternative metal catalysts were developed for catalytic glucose hydrogenation, such as noble metal palladium (Pd), platinum (Pt), ruthenium (Ru), iridium (Ir), and rhodium (Rh), as well as non-noble metal copper (Cu), tungsten (W), cobalt (Co) [26,27,28,29,30]. Among these catalysts, Ru-based catalysts have shown particular promise as the most effective alternative to nicked-based catalysts due to their higher activity, less deactivation and higher selectivity for sorbitol [31,32,33].

To offset the high cost of Ru, catalyst supports have been introduced. These supports not only affect the dispersion of the metal phase but also the stability and selectivity of the catalyst. Examples of catalyst supports include AC, amorphous alloys, zeolites, ordered mesoporous silica (MCM), polymer resins, and metal oxides. Supported catalysts have exhibited enhanced activity in heterogeneous catalysis [19,32,34]. Romero et al. [19] studied Ru catalyst using TiO_2_, C, MCM–48, and MCM–41 as support for the hydrogenation of glucose. The results revealed that the Ru/C catalyst under 5%Ru, 2.5 MPa H_2_, 0.42 h, 120 °C conditions obtained the highest sorbitol yield of 94.8%. AC, in particular, has shown long-term stability under different reaction conditions and can be modified to improve its pore size and electronic structure [1]. Nevertheless, the majority of Ru/C catalysts are prepared using impregnation, which adsorbs the active metal on the support surface, leading to easy deactivation and low recyclability of the catalyst [1]. Therefore, numerous research studies have been conducted to develop catalysts with high performance and recyclability by varying synthesis methodologies, metal precursors, supports, particle size and promoters. Wang et al. [17] prepared Ru–B/NH_2_&CH_3_–MSNSs for the hydrogenation of glucose. Under 4% catalyst ratio, 3 MPa H_2_, 1.33 h, 100 °C conditions, the sorbitol yield was as high as 99%, and the D-glucose conversion was 88% on the tenth cycle.

The aim of this study is to develop a catalyst for the hydrogenation of glucose to sorbitol using amino styrene-co-maleic anhydride polymer-coated active carbon (ASMA@AC) as a support and Ru as a metal ligand. AC offers a high surface area structure with excellent diffusional properties, while the polystyrene structure coordinates with the Ru complex to fully immobilize and confine the active metal, preventing deactivation. The study aims to investigate the conditions of glucose hydrogenation and establish a kinetic equation. The Ru/ASMA@AC and Ru/AC catalysts will be compared in terms of recycle times, and various techniques such as N_2_-adsorption and desorption, X-ray diffraction (XRD), Simultaneous thermogravimetric and differential scanning calorimetry analysis (TGA–DSC), X-ray photoelectron spectroscopy (XPS), Fourier-transform infrared spectroscopy (FTIR), Scanning electron microscope (SEM), Transmission electron microscope (TEM), Inductively coupled plasma-optical emission spectroscopy (ICP–OES) will be used to examine the characteristics of the Ru/ASMA@AC catalyst.

## 2. Results and Discussion

### 2.1. Effects of Catalyst Preparation on Glucose Hydrogenation

The hydrogenation of glucose catalyzed by a supported metal catalyst is the main production method for sorbitol in industry. A series of self-made polymer-coated supported ruthenium catalysts were used for glucose hydrogenation. The catalyst preparation process was investigated by comparing different supports and different ruthenium loading.

**Figure 1:**
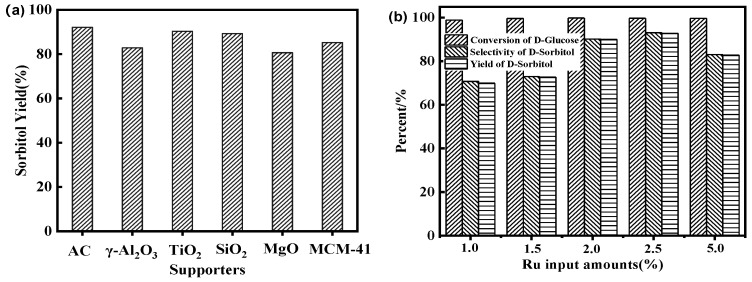
(**a**): effects of different carriers on the yield of sorbitol by hydrogenation of glucose (reaction conditions: 20% glucose aqueous solution 100 g, 2.5 wt.% Ru/ASMA@AC catalyst 1.50 g, reaction temperature 130 °C, hydrogen pressure 4.0 MPa, stirring rate 600 rpm, reaction time 3 h); (**b**): effects of Ru input amounts on the hydrogenation of glucose (reaction conditions: 20% glucose aqueous solution 100 g, Ru/ASMA@AC catalyst 1.50 g, reaction temperature 130 °C, hydrogen pressure 4.0 MPa, stirring rate 600 rpm, reaction time 3 h).

To compare the effects of support types on glucose hydrogenation during catalyst preparation, including γ–Al_2_O_3_, MCM–41, SiO_2_, MgO, and AC were employed [25]. It can be seen from Figure 1a that AC support has the highest activity for glucose hydrogenation, with a sorbitol yield of over 90%. Following AC were TiO_2_, SiO_2_, MCM–41, γ–Al_2_O_3_, and MgO. The specific surface area of oxide carriers such as Al_2_O_3_ and MgO was relatively small, and the number of pores was also small, resulting in uneven metal distribution. In contrast, AC had a high specific surface area and rich pore environment, especially when coated with ASMA with coordinated metal, leading to high dispersion and high catalytic activity.

To investigate the effects of ruthenium loading on glucose hydrogenation, five catalysts with varying ruthenium content were prepared. As illustrated in Figure 1b, when the loading amount is less than 2.5 wt.%, the selectivity of sorbitol improved with increased metal content, as this increased the number of active centers and enhanced catalytic performance. The optimal loading amount was at 2.5 wt.%, with a maximum yield of 92.70% and a final ruthenium content of 0.53%, according to the result of ICP-OES. However, increasing the loading amount to 5 wt.% led to a decrease in sorbitol yield to 82.70%. This might be attributed to the accumulation of a large amount of metal ruthenium on the outer surface of the ASMA@AC support, which weakened the interaction between the ruthenium and support. Furthermore, excessive ruthenium could alter the acidity of the catalyst and lead to reduced catalytic activity during the deep hydrogenation of sorbitol [14]. Additionally, excessive metal content could increase the average size of ruthenium nanoparticles (Ru NPs), reducing the specific surface area of the catalyst and ultimately decreasing its catalytic activity. Therefore, 2.5 wt.% metal ruthenium input amount was the most favorable for glucose hydrogenation. These characteristics contribute to the superior performance of Ru/ASMA@AC in glucose hydrogenation.

### 2.2. Catalyst Characterization

#### 2.2.1. XRD Results

**Figure 2:**
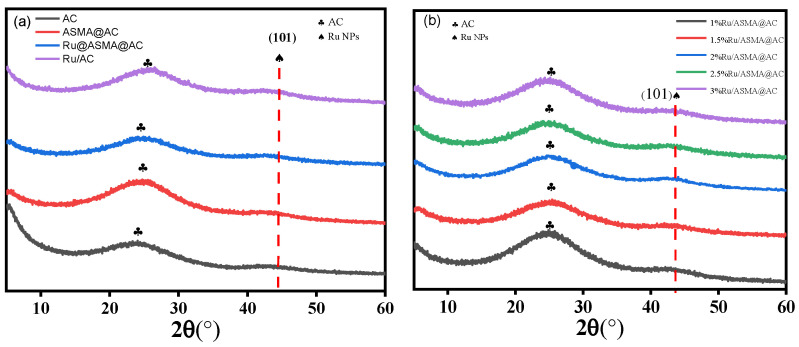
(**a**) XRD pattern of AC, ASMA@AC, Ru/ASMA@AC and Ru/AC; (**b**) XRD patterns of Ru/ASMA@AC catalysts with various metal concentrations.

To determine whether AC exhibits crystallization before and after polymerization, XRD diagrams of metal ruthenium before and after ruthenium loading, as well as various ruthenium loading, were analyzed (Figure 2a,b). The fuzzy peak in the XRD spectrum of Figure 2a indicates the low crystallinity of macroporous activated carbon and the low crystallinity when encapsulated by ASMA in the ASMA@AC sample. The characteristic peak of AC is observed at approximately 24.7°, while the diffraction peak becomes dispersed after ruthenium loading. In Figure 2b, it is observed that when the loading amount of ruthenium was less than 3 wt.%, it was typically covered by the carrier, and no obvious ruthenium diffraction peak can be observed. This experiment used a loading amount of 2.5 wt.% of metal ruthenium, which resulted in only a small peak at approximately 43.6° for Ru/ASMA@AC and Ru/AC (JCPDSNo. 06-0663) [18], corresponding to the 101 crystal plane of Ru(0) [35]. This indicates that Ru NPs were successfully loaded into both the ASMA@AC and the AC carrier.

#### 2.2.2. TGA–DSC Results

**Figure 3:**
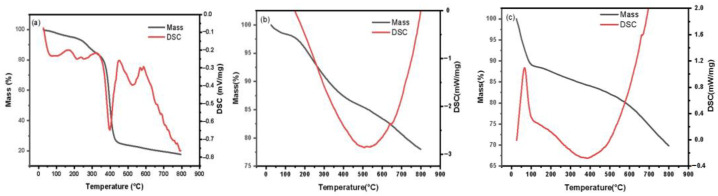
TGA-DSC curves (**a**) Ru/ASMA; (**b**) Ru/ASMA@AC; (**c**) Ru/AC.

The thermogravimetric properties of Ru/AC, Ru/ASMA and Ru/ASMA@AC are shown in Figure 3. All three samples showed slight weight loss near 100 °C due to the vaporization of water or organic solvents present in the catalyst. In Figure 3a, an obvious endothermic peak at around 398 °C was observed, which corresponds to the decomposition of ASMA, as shown by the endothermic peak near 504 °C in Figure 3b. Compared to the AC as the catalyst core, the thermal decomposition temperature of the polymer increased by 106 °C, indicating that the AC alleviates the thermal shock and the carrier ASMA@AC has better thermal stability. The AC without polymer encapsulation is shown in Figure 3c, and some mass loss occurred at around 400 °C, further supporting the improved thermal stability of ASMA@AC compared to pure AC.

#### 2.2.3. N_2_-Adsorption and Desorption Results

**Figure 4:**
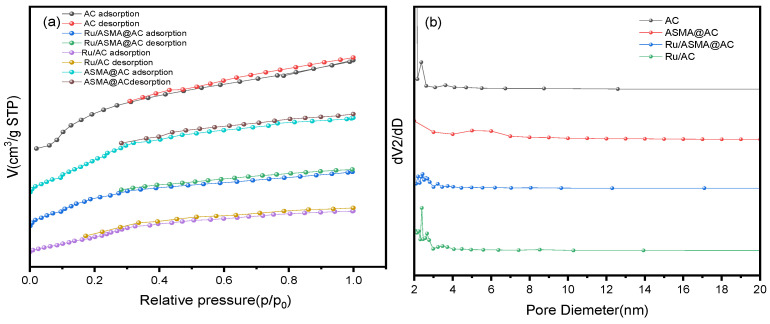
(**a**) N_2_ isotherm adsorption and desorption curve of AC before and after polymerization and metal impregnation; (**b**) Pore size distribution diagram before and after AC polymerization and metal coordinated.

The N_2_-adsorption and desorption results are presented in Figure 4a and Table 1, which show the changes in the specific surface area of AC before and after polymerization and metal loading. The adsorption and desorption isotherms of all samples were type I and type IV isotherms, respectively, indicating the presence of both micropores and mesopores in the samples [35]. The specific surface area of AC decreased from 1064.83 m^2^/g to 507.99 m^2^/g after polymerization, possibly due to the decrease in adsorption capacity caused by the high fluidity of the polymer. The hysteresis ring was significantly reduced after Ru was coordinated onto the ASMA@AC, indicating a decrease in the mesoporous amount of the carrier and a decrease in the specific surface area. The specific surface area of Ru/AC was 602.13 m^2^/g, while that of Ru/ASMA@AC catalyst was 431.60 m^2^/g, which can be attributed to the existence of ASMA. Figure 4b shows that the samples maintained a mesoporous structure (2~50 nm) even after polymerization and metal loading, revealing that Ru NPs were closely coordinated with the outer surface through −NH_2_ and −OH on the polymer, which can inhibit Ru NPs from falling off from the shell [36].

#### 2.2.4. XPS Results

**Figure 5:**
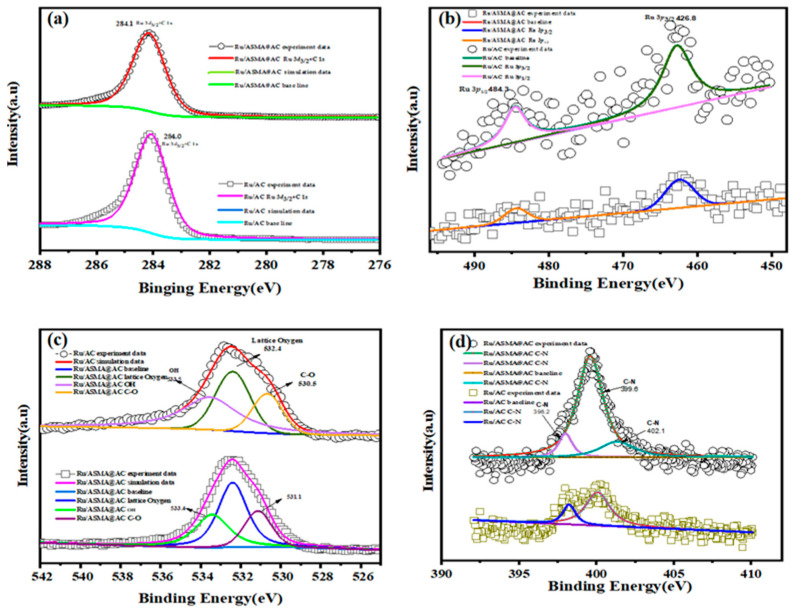
XPS spectra of (**a**) Ru 3*d*; (**b**) Ru 3*p*; (**c**) O 1*s;* (**d**) N 1*s* for the 2.5 wt.% Ru/AC and 2.5 wt.% Ru/ASMA@AC.

To study the relationship between electron donor groups and Ru active sites on the carrier surface, XPS analysis was performed. Figure 5a–d displays the spectral regions of Ru 3D, Ru 3P, O 1s, and N1s for Ru/ASMA@AC and Ru/AC. In Figure 5a, typical XPS peaks appeared at 284.1 eV and 284.0 eV, which were close to the theoretical value of 284.0 eV of Ru^0^/ASMA@AC and Ru/AC catalysts that were fully reduced [35]. This suggests that the catalyst was effectively reduced during the preparation process. Figure 5b illustrates the binding energies of the two catalysts at 484.3 eV and 462.8 eV, which were slightly higher than the theoretical values of Ru (0), 484.0 eV and 462.0 eV. This confirmed that the catalyst contained a small amount of high valence ruox and Ru, confirming that Ru^3+^ was mainly reduced to Ru (0) [37]. Moreover, in Figure 5d, an obvious binding energy was observed in the N 1s orbit of Ru/ASMA@AC, with three portions corresponding to C-N (398.2 eV), C-N (399.6 eV), and C-N (402.1 eV) [38,39,40]. This suggests that N species were present in the ASMA and transferred charge to the coordinated active sites. The active sites, enriched with electrons, acted as a bridge to facilitate the transfer of electrons. In contrast, the N1S in Ru/AC may be attributed to the impurities in the preparation process of AC. The strong electronic interaction between the ASMA polymer support and the active sites enables N to act as an electron donor, similar to an electron promoter, as Zhang et al. [41] reported. The C-N (399.6 eV) of Ru/ASMA@AC indicates that N species in ASMA transfer charge to the coordinated active sites. The results confirm that ASMA polymer was successfully wrapped on the AC surface and played a crucial role in the electronic interaction of the catalyst.

#### 2.2.5. FTIR Results

**Figure 6:**
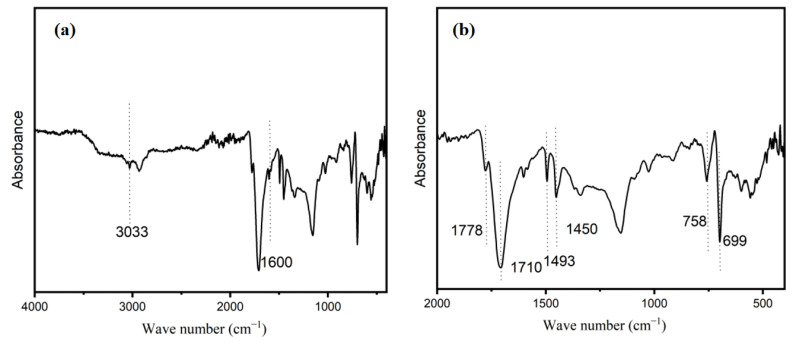
FT-IR diagram of polymer shell without AC in transmission mode. (**a**) 4000–400 cm^−1^; (**b**) 2000~500 cm^−1^.

To determine the main chemical composition of the polymer ASMA, FTIR spectroscopy was conducted. As shown in Figure 6, the broad and weak peaks at around 3033 cm^−1^ may be attributed to the hydroxyl (−OH) or amino (−NH_2_) groups on ASMA. Additionally, the peaks at 1600 and 1493 cm^−1^ correspond to the skeleton vibration of the benzene ring [42]. While the bands at 699 and 758 cm^−1^ are caused by the out-of-plane bending vibration of C–H in the benzene ring [43]. The bands near 1710 and 1778 cm^−1^ are attributed to the symmetric and asymmetric stretching vibrations of the C=O group in the maleic anhydride unit, respectively. The presence of para-substituted benzene can be detected by a dielectric peak at 1450 cm^−1^ [36]. Overall, the results of infrared spectroscopy indicate that the main chemical composition of the self-made copolymer in this experiment is styrene and maleic anhydride.

#### 2.2.6. SEM Results

**Figure 7:**
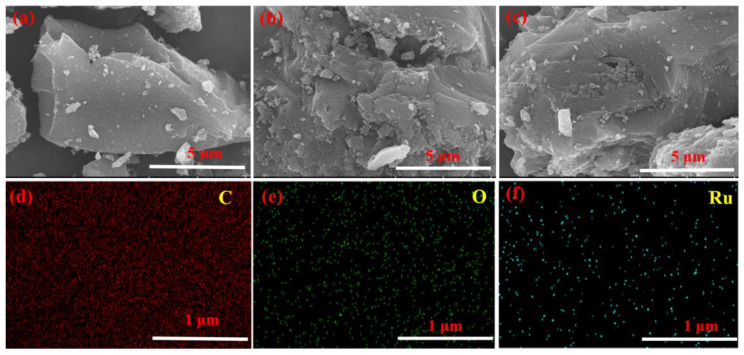
(**a**) SEM image of blank activated carbon; (**b**) SEM image of ASMA@AC; (**c**) SEM image of 2.5 wt.% Ru/ASMA@AC; (**d**) C element distribution; (**e**) O element distribution; (**f**) Ru element distribution.

To investigate the impact of polymerization on AC, the SEM was utilized to study the morphology of AC support. As depicted in Figure 7a, the blank AC had clear and abundant pores. After being wrapped with ASMA, as shown in Figure 7b, the pore structure of the @ASMAAC carrier remained unchanged. As shown in Figure 7c, after 2.5 wt.%, Ru was coordinated with the ASMA@AC, Ru/ASMA@AC catalyst also maintained the typical characteristics of AC support. These results implied that both the polymerization and the coordination of the catalyst do not affect the morphological characteristics of the AC support. Moreover, the rich microporous structure of the AC was conducive to the even distribution of Ru nanoparticles.

#### 2.2.7. TEM Results

**Figure 8:**
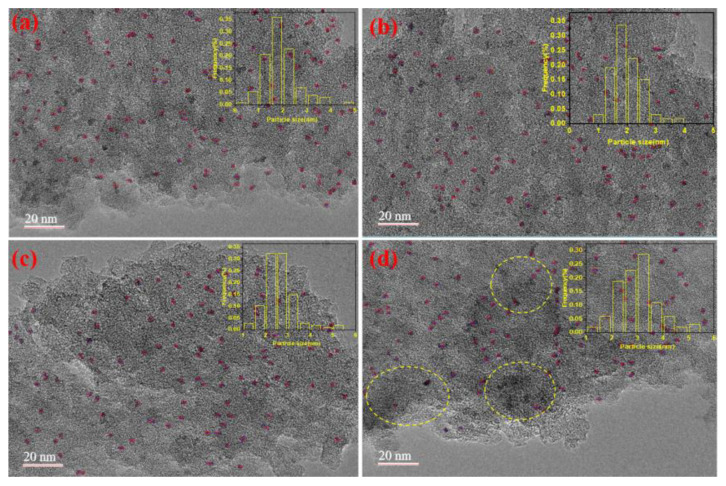
(**a**) TEM image and Ru NPs size distribution of 2.5 wt.% Ru/ASMA@AC; (**b**) TEM image and Ru NPs size distribution of 2.5 wt.% Ru/ASMA@AC after five reactions; (**c**) TEM image and Ru NPs size distribution of 2.5 wt.% Ru/AC; (**d**) TEM image and Ru NPs size distribution of 2.5 wt.% Ru/AC after five reactions.

To investigate the anchoring effect of the polymer on ruthenium, as well as the state and distribution of ruthenium in the 2.5 wt.% Ru/ASMA@AC and 2.5 wt.% Ru/AC before and after the reaction, TEM analysis was conducted. Figure 8a–d illustrates the TEM images and particle size distribution of Ru NPs in two different catalyst systems, 2.5 wt.% Ru/ASMA@AC and 2.5 wt.% Ru/AC, before and after five reaction cycles.

In Figure 8a, it was observed that ruthenium metal particles were evenly distributed on the carrier within Ru/ASMA@AC. This suggests that the ASMA was evenly distributed on the surface of the AC, forming a network cross-linking structure with a certain pore structure. This structure was beneficial for the coordination of ruthenium metal to the carrier and could reduce the falling deactivation of Ru. The average particle size of metal ruthenium was 1.90 nm, and no agglomeration was observed. In Figure 8b, after five reaction cycles, the Ru/ASMA@AC remained at a content of 0.53%, and the average particle size of metal ruthenium was 2.0 nm. The Ru NPs did not agglomerate, and high catalytic performance was maintained. This may be due to the N complexation with Ru particles, which blocked the path of Ru NPs aggregation, preventing further particle growth. This finding is consistent with Kim et al.’s study, in which they used a Ru-vertically standing anchored planar 2D catalyst with vertically standing pyrazine N anchors to block the path of Ru NPs aggregation [38]. In Figure 8c, Ru/AC showed that the Ru NPs were also distributed evenly on the surface of the AC, and the average particle size of metal ruthenium was 2.65 nm. However, after five cycles of reaction, the Ru was reunited. The highlighted portion within the yellow dashed circle indicates that significant agglomeration occurred during catalysis, resulting in the average particle size of metal ruthenium being 3.02 nm, and the particles were easy to fall off, as seen in Figure 8d. The initial ruthenium content of Ru/AC was 0.54%, according to the result of ICP-OES, but after five reaction cycles, it was only content 0.38%, revealing the fall off of the Ru NPs.

Therefore, the self-made carrier was helpful for the uniform dispersion of Ru NPs, resulting in ultrafine nanometal active centers that were conducive to hydrogenation. This study highlights the importance of the carrier in maintaining the stability and activity of the catalyst.

### 2.3. Ru/ASMA@AC Catalyst Ability

In order to investigate the conditions for the glucose hydrogenation reaction using Ru/ASMA@AC catalyst, various effects were examined, including catalyst dosage, substrate concentration, reaction temperature, stirrer speed, H_2_ pressure and reaction time.

**Figure 9:**
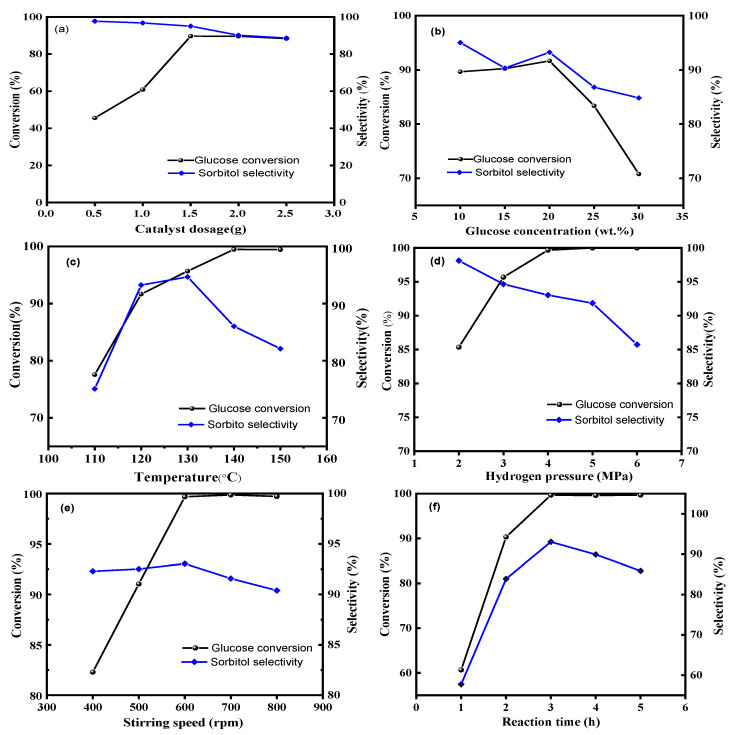
Effects of reaction condition on the yield of sorbitol by hydrogenation of glucose (**a**): Ru/ASMA@AC catalyst dosage (120 °C, 10% glucose concentration, 3 MPa, 600 rpm, 3 h); (**b**): glucose concentration (1.5 g catalyst, 120 °C, 3.0 MPa, 600 rpm, 3 h); (**c**): reaction temperature (1.5 g catalyst, 20% glucose concentration, 3.0 MPa, 600 rpm, 3 h); (**d**): hydrogen pressure (1.5 g catalyst, 20% glucose concentration, 130 °C, 600 rpm, 3 h); (**e**): stirring speed, (1.5 g catalyst, 20% glucose concentration, 130 °C, 4.0 MPa, 3 h); (**f**): reaction time (1.5 g catalyst, 20% glucose concentration, 130 °C, 4.0 MPa, 600 rpm)).

The relationship between catalyst dosage, sorbitol yield, and glucose conversion is illustrated in Figure 9a. Sorbitol yield increased as catalyst dosage increased. The maximum conversion of glucose and selectivity of sorbitol were achieved with a 1.5 g catalyst, with values of 89.66% and 95.04%, respectively. Subsequently, increasing the catalyst amount had little effect on yield, with glucose almost completely converted. In this experiment, the reactant glucose amount was fixed, and high conversion was not observed even with an excessive catalyst. Therefore, a 1.5 g catalyst amount was selected.

To investigate the effect of glucose concentration on the catalytic reaction, various concentrations of substrate were prepared for reaction with fixed amounts of catalyst. As shown in Figure 9b, increasing glucose concentration from 10 wt.% to 20 wt.% improved substrate conversion and product selectivity. However, upon further increasing glucose concentration, substrate conversion decreased from 91.66% to 93.25%, along with decreasing sorbitol selectivity. The maximum conversion of glucose and selectivity of sorbitol were reached when the glucose concentration was 20%. This phenomenon could be attributed to the difference in the adsorption of glucose on the Ru/ASMA@AC catalyst [15]. At lower concentrations, the hydrogenation rate increased with concentration as surface adsorption did not reach saturation. When the glucose concentration increased to 20%, the adsorption reached saturation. When the substrate concentration continued to increase, glucose adsorbed on the catalyst was supersaturated, and part of the substrate could not be transformed, resulting in the reduction of glucose conversion and the selectivity of sorbitol. Therefore, based on the conversion of glucose and the selectivity of sorbitol, the optimal glucose concentration of 20 wt.% was selected as the subsequent study.

Figure 9c illustrates the impact of temperature on sorbitol selectivity during glucose conversion. When the temperature rose from 110 to 130 °C, the conversion and selectivity of glucose increased sharply. When the reaction temperature reached 130 °C, the glucose reaction solution was almost entirely consumed, resulting in a conversion rate of 95.68%. At this temperature, the reaction also reached maximum selectivity, with a selectivity rate of 94.66%. When the temperature rose from 130 to 150 °C, the conversion rate of glucose remained relatively constant while the selectivity rate decreased sharply. In particular, high temperatures had a significant impact on the selectivity of sorbitol, as they could lead to undesirable side reactions, including glucose coking, glucose isomerization, and sorbitol decomposition [44,45,46]. Excessively high temperatures could also generate unwanted byproducts, such as fructose and mannitol, as shown in Figure 9c. Therefore, a temperature of 130 °C was found to be the optimal reaction temperature.

Reaction pressure is an important parameter in the hydrocracking process [47]. An increase in pressure resulted in a corresponding increase in the driving force and diffusion rate of hydrogen, leading to improved substrate conversion. Pressure levels of 2.0 MPa, 3.0 MPa, 4.0 MPa, 5.0 MPa, and 6.0 MPa were selected, respectively. As shown in Figure 9d, with the hydrogen pressure of 2.0 MPa and 3.0 MPa, the conversion rate of glucose increased from 75.40% to 90.0%, and the selectivity of sorbitol also increased. This effect was due to the increased concentration of dissolved hydrogen in water at higher pressures, which causes greater adsorption of glucose on the surface of the Ru/ASMA@AC catalyst. At a reaction pressure of 4 MPa, the highest sorbitol yield was obtained, with the conversion of glucose of 99.68% and the selectivity of sorbitol of 93.04%, due to the maximum solubility of hydrogen in water and the resulting optimal catalytic effect. However, when the pressure reached above 4.0 MPa, glucose reaction conversion was essentially complete, and the selectivity of sorbitol did not change significantly. This might be attributed to the high concentration of hydrogen dissolved in water, leading to saturation and limiting the impact of further increases in hydrogen pressure. Moreover, under high stirring rates, mass transfer and the diffusion of glucose and hydrogen might be impeded while simultaneously damaging the catalyst walls. As a result, an optimum hydrogen pressure of 4.0 MPa was selected.

As can be seen from Figure 9e, the conversion rate of glucose was only 82.29% at a lower stirring speed of 400 rpm. This was because the three-phase system was not evenly dispersed at lower speeds, and the sorbitol generated could not be desorbed from the catalyst support in time, resulting in a low yield of sorbitol. By gradually increasing the stirring rate, the yield of sorbitol significantly increased. Increasing the hydrogen pressure could increase the solubility of hydrogen in water and accelerate the hydrogenation rate of glucose. When the stirring rate was increased to 600 rpm, the yield of sorbitol reached 92.74%. At a higher stirring rate, the catalyst could be evenly dispersed in the solution, allowing for more efficient diffusion reactions between glucose and the catalyst. Additionally, the generated sorbitol could quickly fall off the catalyst surface. However, excessively high stirring rates could also cause the catalyst to stick to the reactor wall, resulting in low catalyst utilization. Therefore, a stirring rate of 600 rpm was optimal in terms of both economic cost and safety.

Optimum reaction times exist for each chemical reaction, and any duration deviation from that can impact the selectivity of the reaction. When investigating the reaction time, one must consider attaining efficient reaction efficiency while reducing the occurrence of side reactions. Simultaneously, it is also vital to consider equipment occupancy time to optimize costs. To determine the optimal reaction time, five different time points were selected for analysis. As can be observed in Figure 9f, the conversion rate of glucose increased thoroughly with the prolonged reaction time. When the reaction time reached 3 h, glucose was entirely converted, and the conversion rate reached its maximum value of 99.68%. In the initial stage of hydrogenation, glucose was converted to yield sorbitol, which attained maximum yield at 3 h. Over time, the yield of sorbitol diminished, likely due to isomerization to mannitol. Moreover, a small portion of sorbitol compounds degraded into lower alcohols, including ethylene glycol, glycerol, propylene glycol, and other C4–C6 polyols. Therefore, the yield of sorbitol was 92.74% under the conditions of catalyst dosage of 1.50 g, 20 wt.% glucose aqueous solution of 100 g, reaction temperature of 130 °C, hydrogen pressure of 4.0 MPa and stirring speed of 600 rpm.

The results were consistent with previous research studies. Guo et al. [14] employed Ru/ZSM-5 catalyst for the conversion of D-glucose, achieving as high as 99.6% conversion with D-sorbitol selectivity reaching 99.2%, at 200 °C, 4.0 MPa H_2_, catalyst 0.5 g, 25 wt.% D-glucose aqueous solution. Although the D-glucose concentration was higher than that used in this work, the reaction temperature was much higher. Dabbawala et al. [48] studied Ru/polymer catalyst under 5%Ru, 5%glucose concentration, 5.5 MPaH_2_, 1 h, 100 °C conditions and obtained a sorbitol yield of 67.5%. Lazaridis et al. [49] used Ru/AC 3%Ru, 1.6 MPa H_2_, 3 h, 180 °C conditions to obtain a sorbitol yield of 89% while using Pt/AC required more Pt loading to achieve the same sorbitol yield.

### 2.4. Study on Catalytic Reaction Kinetics

**Figure 10:**
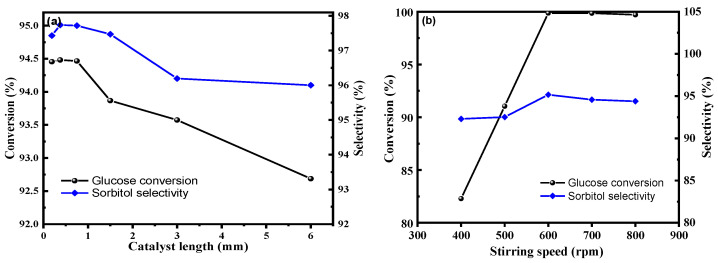
(**a**): the influence of internal diffusion on the reaction (reaction conditions: 20% glucose aqueous solution 100 g, Ru/ASMA@AC catalyst 1.50 g, reaction temperature 130 °C, hydrogen pressure 4.0 MPa, stirring rate 600 rpm, reaction time 3 h), (**b**): the influence of external diffusion on the reaction (reaction conditions: 20% glucose aqueous solution 100 g, 0.375 mm length Ru/ASMA@AC catalyst 1.50 g, reaction temperature 130 °C, hydrogen pressure 4.0 MPa, reaction time 3 h).

Chemical kinetic equations are essential in evaluating reaction rates and reaction complexities, serving as fundamental premises in large-scale chemical production techniques. To mitigate the influence of internal diffusion, catalytic reactions were performed in a columnar shape at intervals [50]. As presented in Figure 10a, the optimal catalyst length was found to be 0.375 mm. Figure 10b illustrates that the best reaction efficiency was achieved at a stirring speed of 600 rpm, removing the influence of mass transfer on the reaction. Based on these findings, the macro kinetics of the reaction process were studied to provide a theoretical basis for industrial-scale production scale-up.

**Figure 11:**
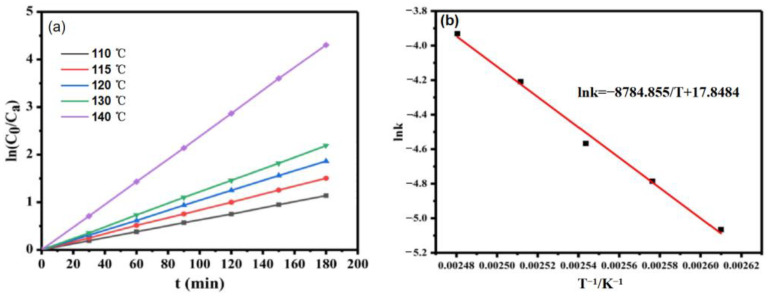
(**a**): the relationship between time and ln(C_0_/Ca) at different temperatures (reaction conditions: 20 wt.% glucose aqueous solution, Ru/ASMA@AC catalyst 1.5 g, reaction temperature 130 °C, hydrogen pressure 4 MPa, stirring rate 600 rpm, reaction time 3 h), (**b**): the relationship between T^−1^ and lnk (reaction conditions: 20 wt.% glucose aqueous solution, Ru/ASMA@AC catalyst 1.50 g, reaction temperature 130 °C, hydrogen pressure 4 MPa, stirring rate 600 rpm, reaction time 3 h).

The plot of ln(C_0_/C_a_) versus time was linear, as shown in Figure 11a. At the same temperature, it is obvious that time had a linear relationship with ln(C_0_/C_a_), indicating that the hydrogenation of glucose catalyzed by Ru/ASMA@AC followed the first-order kinetics with respect to glucose concentration. Mishra et al. [25] obtained similar results.

For the first-order reaction, the activation energy can directly reflect the difficulty of the chemical reaction [15,19]. According to Arrhenius’s equation $\;\mathrm{lnk}=-\frac{E_{a}}{\mathrm{RT}}+\mathrm{lnA}\;$, the activation energy can be obtained by the relationship between the reaction rate constant obtained at different temperatures [25].

As shown in Figure 11b, linear regression is conducted between *lnk* and *T*^−1^ to obtain the equation *lnk* = −8784.855/*T* + 17.8404 (*R*^2^ = 0.9918), with an activation energy *E_a_* = 73.04 kJ/mol, and *A* of 0.03 m^3^(kmol·s). At this point, the kinetic equation is −r_A_= 0.03 exp (−73.04/(RT))Ca, indicating that the catalyst has high efficiency and facilitates an easy reaction. According to Romero’s research, the activation energy values obtained for Ni/MCM-48 (36 KJ mol^−1^) and Ru:Ni/MCM-48 (0.45; 70 KJ mol^−1^), which indicates that the reaction rate was controlled by the kinetics of the metal surface [18]. Based on Mishra’s research, the apparent activation energy amounts to 24~49 kJ/mol, depending on the initial D-glucose concentration and density and viscosity of the solution [25].

### 2.5. Catalyst Reuse Test

**Figure 12:**
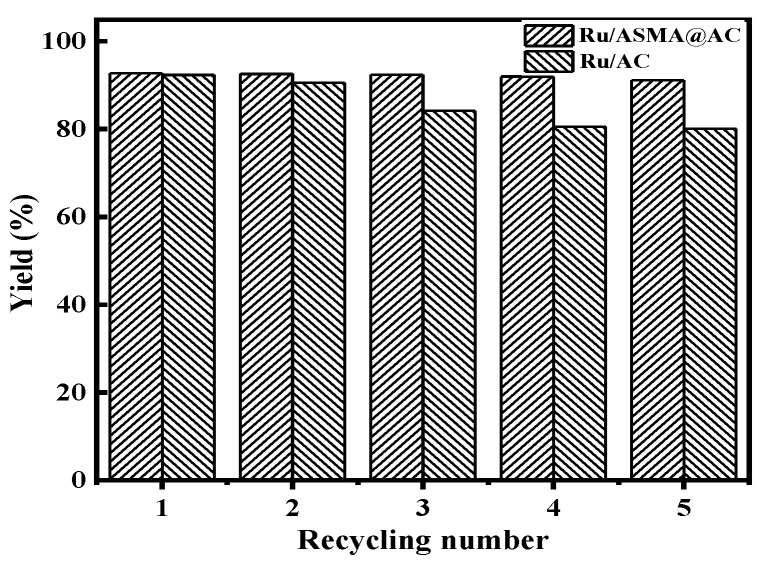
Catalyst recycle number test (reaction conditions: 20% glucose aqueous solution 100 g, Ru/ASMA@AC catalyst 1.5 g, reaction temperature 130 °C, hydrogen pressure 4 MPa, stirring speed 600 rpm, reaction time 3 h).

To investigate the stability of the catalyst, 1.50 g of Ru/ASMA@AC or a Ru/AC catalyst and 20 wt.% glucose aqueous solution was placed in a 150 mL batch reactor with a reaction temperature of 130 °C, hydrogen pressure of 4 MPa, rotating speed of 600 rpm and reaction time of 3 h. After each reaction, the catalyst was separated from the solution, washed with deionized water and dried overnight at 110 °C. The results of repeated glucose hydrogenation with the catalyst are shown in Figure 12.

The results in Figure 12 demonstrate the reaction effect of the Ru/AC and Ru/ASMA@AC catalysts. The yield of sorbitol decreased from 92.36 to 84.18% after three reactions when using the Ru/AC catalyst. After five reactions, the sorbitol yield was further reduced to only 80.1%. In contrast, the Ru/ASMA@AC catalyst maintained a high conversion of glucose and high selectivity of sorbitol, resulting in a sorbitol yield of 92.41% after the third cycle with no significant decrease. Moreover, the sorbitol yield was 91.18% after five reactions, indicating that there was no significant loss of sorbitol yield after five reactions of the Ru/ ASMA@AC catalyst. These results suggest that the Ru/ASMA@AC catalyst has excellent stability and reusability, making it more suitable for practical applications than the Ru/AC catalyst. ICP-OES analysis confirmed that the Ru/ASMA@AC catalyst still contained 0.53% content of Ru after five reactions, while the content of Ru in the Ru/AC catalyst was only 0.38%. This may be due to the stable complex coordination between nano metal ruthenium and polymer, which made it difficult for Ru to detach from the carrier [41]. Therefore, the Ru/ASMA@AC catalyst has a higher potential for process application value than Ru/AC. Similarly, Silva et al. [1] used a RuNb_2_O_5_ catalyst and obtained a selectivity of 98% for sorbitol and a glucose conversion rate of 82% after the third cycle, decreasing by 3% and 1%, respectively. Guo et al. [14] used Ru/ZSM–5 catalyst prepared by the conventional impregnation method for five cycles and observed that the decrease in glucose conversion was from 99.7% to 89.2%. They attributed this to the regeneration method of the catalyst employed.

### 2.6. Reaction Mechanism

**Figure 13:**
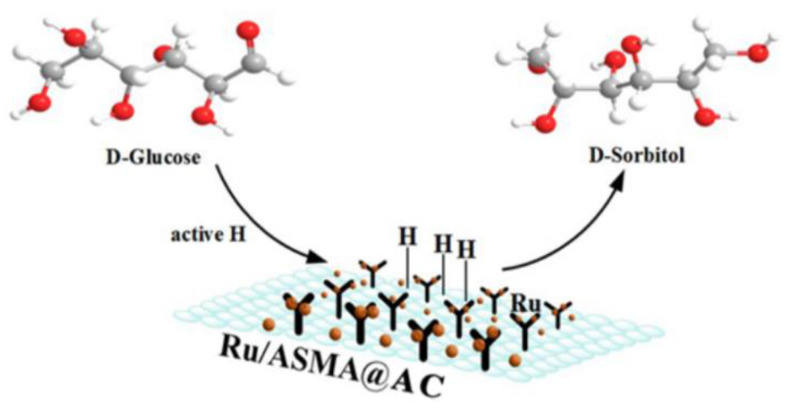
The hydrogenation route of glucose to sorbitol catalyzed by Ru/ASMA@AC catalyst.

In this study, styrene maleic anhydride supported on activated carbon was used as the catalyst carrier, and the maleic anhydride unit had high reactivity to surface hydroxyl [51]. Through the direct induction of external hydroxyl groups, ASMA polymers can be tightly grafted onto the surface of activated carbon. In the process of impregnating and coordinating metal ruthenium, the outermost −NH_2_ and −OH groups play a key role as nucleophilic targets to attract more dissociative Ru cations to gather outward on the surface, respectively. After activation with hydrogen, Ru NPs can form a stable complex with the polymer, and highly dispersed Ru can promote the hydrogenation of glucose to sorbitol [35]. Glucose hydrogenation is a three-phase reaction of gas, liquid and solid, as shown in Figure 13.

In the hydrogenation process, Glucose hydrogenation is a three-phase reaction of gas, liquid and solid. H_2_ contacts with metal Ru to form active hydrogen, which further attacks the carbonyl of glucose to form sorbitol. H_2_ is first adsorbed on the uniformly dispersed Ru catalytic site to form active hydrogen. When combined with glucose molecules, the active hydrogen will immediately attack the carbonyl group of the reactant [35], resulting in the formation of target sorbitol, which was then desorbed into the mixed solution. The presence of external −OH and −NH_2_ groups on ASMA gave a strong hydrogen bond, which helped to bind the Ru NPs more firmly and evenly dispersed them on the ASMA@AC carrier than the pure van der Waals force between Ru NPs and AC particles. The highly dispersed Ru could accelerate the hydrogenation of glucose to sorbitol rather than the side reactions such as isomerization, carbonization, and cleavage reactions [34]. Moreover, the strong coordination between Ru NPs and ASMA coated with AC could further improve the recoverability of the catalyst. The amino and hydroxyl groups on the surface of the Ru/ASMA@AC catalyst prepared in this subject act as nucleophilic targets, which coordinated and confined more Ru than only the impregnation process of the Ru/AC catalyst. Glucose hydrogenation is a three-phase reaction of gas, liquid and solid. H_2_ contacts with metal Ru to form active hydrogen, which further attacks the carbonyl of glucose to form sorbitol.

## 3. Materials and Methods

### 3.1. Materials

Columnar particle-activated carbon was purchased from Fujian Xinsen Carbon Industry Co., Ltd. (Shaowu, China), and Maleic anhydride and divinylbenzene (55%) were purchased from Aladdin Holding Group Co. Ltd. (Shanghai, China). All the other chemicals were purchased from Sinopharm Chemical Reagent Company (Shanghai, China). All the reagents were of analytical grade and used as received without further purification.

### 3.2. Catalysts Preparation

**Figure 14:**
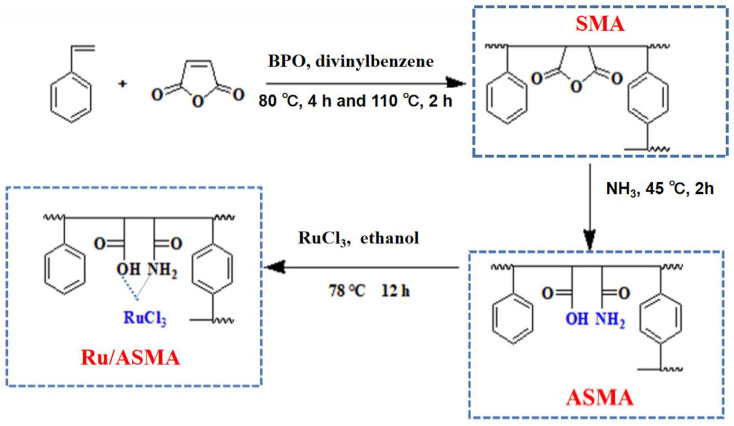
The synthesis process of catalyst polymer coordinated with ruthenium.

The columnar particle AC was soaked in deionized water and ultrasonically cleaned using a cleaning machine (Kunshan Ultrasonic Instrument Co., Ltd., Kunshan, China) to remove dust from the channels. The AC was then dried and kept aside. The catalyst complex precursor, SMA, was prepared as follows: the mass ratio of styrene, benzoyl peroxide, divinylbenzene, and maleic anhydride was 200:1:20:300, where styrene and maleic anhydride were the polymerization monomers, and trace amounts of divinylbenzene and benzoyl peroxide (BPO) were used as a crosslinking agent and an initiator, respectively. The pretreated AC was drained after being soaked in the polymerization solution for 4 h. The polymerized activated carbon was purged in an oven and programmed at 80 °C for 4 h and 110 °C for 2 h to fix the polymer onto the activated carbon. Considering the maleic anhydride is breakable, the SMA/AC was ammonolized in 45 °C aqueous ammonia solution for 2 h, neutralized, and then dried to obtain ASMA@AC. Afterward, ASMA@AC was impregnated in ruthenium trichloride ethanol solution and refluxed at 78 °C for 12 h [34,35]. Ruthenium trichloride was used as the active precursor to obtain Ru/ASMA@AC. The synthesis process of catalyst polymer coordinated with ruthenium was shown in Figure 14. Moreover, the catalysts on γ–Al_2_O_3_, TiO_2_, SiO_2_, MCM–41, and MgO were prepared the same way. Finally, the obtained catalysts were reduced at 120 °C for 5 h under a hydrogen atmosphere of 5.0 MPa in an autoclave and washed with deionized water 3 times, and then vacuum dried at 105 °C for use. To investigate the effect of the polymer on the Ru catalyst, the AC carrier was directly impregnated in ruthenium trichloride ethanol solution and refluxed at 78 °C for 12 h, and then the Ru/AC was obtained. The subsequent reduction steps were the same as mentioned above.

### 3.3. Conversion of Glucose into Sorbitol

The reaction to convert glucose into sorbitol was performed in a 150 mL stainless autoclave under vigorous stirring. The autoclave was sealed and ventilated after preparing different concentrations of glucose aqueous solution and adding them into the autoclave along with varying catalyst dosages. The air was then replaced with hydrogen more than 3 times, and the H_2_ pressure was adjusted to a certain pressure. After starting the agitator at 200 r/min, the temperature was increased to the target temperature and the agitator speed was adjusted to a settled speed while the reaction was timed. Once the preset time was reached, the reaction was allowed to cool, and the catalyst was filtered and washed. The filtered product was vacuum dried at 105 °C for 2 h and used for the subsequent processes. Analyzing the components of the obtained filtrate was performed by using high-performance liquid chromatography (HPLC; LC–20AD, SHIMADZU, Japan). The liquid chromatographic column used was SC1011 with an examining temperature of 80 °C and the mobile phase being ultrapure water with a flow rate of 1 mL/min. The samples were determined in triplicate, and the average value was taken. The glucose conversion, sorbitol selectivity, and sorbitol yield equations are listed in Equations (1)–(3) [1,18].

(1) $$\mathrm{Glucose}\;\mathrm{conversion}\left(\%\right)=\frac{{\mathrm{mols}\;\mathrm{glucose}}_{\mathrm{reacted}}\;}{{\mathrm{mols}\;\mathrm{glucose}}_{\mathrm{initial}}}\times 100\%$$

(2) $$\mathrm{Sorbitol}\;\mathrm{selectivity}\left(\%\right)=\frac{{\mathrm{mols}\;\mathrm{sorbital}}_{}\;}{{\mathrm{mols}\;\mathrm{glucose}}_{\mathrm{reacted}}}\times 100\%$$

(3) $$\mathrm{Sorbitol}\;\mathrm{yield}\left(\%\right)=\frac{\mathrm{Glucose}\;\mathrm{conversion}\times \mathrm{sorbitol}\;\mathrm{yield}}{100}$$

### 3.4. Catalyst Characterization

#### 3.4.1. XRD

For determining the samples in this experiment, the Rigaku Smartlab TM 9 kW diffractometer (Rigaku, Tokyo, Japan) equipped with Cu α Ray as the excitation source (λ= 1.542 Å) was used. The voltage was 40 kV, the current was 100 mA, and the angle range was 10°~60°.

#### 3.4.2. SEM

The samples were ground into powder in a mortar, a certain quality of samples was weighed an appropriate amount of ethanol solution was added to a prepared mixed solution, which was then dispersed evenly by ultrasound. The samples were sprayed with gold using an MSP–2S ion sputtering instrument (Hitachi, Hitachi, Japan) to increase their conductivity and then imaged under a working voltage of 200 kV of the SU8010 scanning electron microscope (Hitachi, Hitachi, Japan).

#### 3.4.3. TEM

A few milligrams of catalyst powders were dispersed in a 2 mL ethanol solution by ultrasound and then dropped on a copper mesh covered with 300 mesh porous carbon film. Images were taken under an accelerating voltage of 200 kV by JEM 2100F microscope (Jeol, Akishima, Japan). The average particle size of the metal was calculated using the statistical average method based on the images.

#### 3.4.4. N_2_-Adsorption and Desorption

After being vacuum degassed at 200 °C to remove surface impurities and dust, the samples were subjected to nitrogen adsorption at −196 °C using the JW-BK122W specific surface and pore size adsorption instrument (Jingwei Gaobo, Beijing, China). The specific surface area (SSA) and porosity were calculated using the Brunauer–Emmett–Teller (BET) and Barrett-Joyner-Halenda (BJH) methods, respectively.

#### 3.4.5. XPS

Under ultra vacuum conditions, the monochromatic K-Alpha spectrometer (Thermo Fisher Scientific, Waltham, MA, USA) was used. The samples were first pressed into a 1–13 mm sheet and then placed into the XPS analysis chamber for measurement. The test voltage was 12 kV, and the working current was 6 mA. All spectra were corrected for the binding energy with the C 1s peak (284.8 eV) as the reference value.

#### 3.4.6. FTIR

FTIR (Nicolet iS5, Thermo Fisher Scientific, Waltham, MA, USA) was employed to detect the changes in the functional groups of the obtained polymer. The sample to be tested was vacuum dried at 50 °C for 4 h to remove the surface moisture of the sample. A certain mass of the sample was weighed and mixed with KBr, and placed in an infrared tablet mold. The tablet was pressed under a pressure of 0.4 MPa for infrared detection, with a testing range of 400–4000 cm^−1^.

#### 3.4.7. TGA-DSC

Thermal analysis measures the changes in physical properties of materials with temperature under programmed temperature control, such as melting, evaporation, and sublimation. It is widely used in measuring the degradation of metal complexes and the hydrolysis of substances. The STA 2500 synchronous thermal analyzer (NETZSCH, Selb, Germany). was used in this experiment. The sample was carried in an aluminum oxide pot, and the testing process was completed under a nitrogen atmosphere at a heating rate of 10 °C/min, with a temperature range of 30–800 °C.

#### 3.4.8. ICP-OES

The 720 ES model (Agilent, Palo Alto, CA, USA) was used to determine the content of Ru in the sample. A 100 mg catalyst sample was ablated in 10 mL aqua regia solution for 48 h. The solution was then left to stand until the catalyst was completely dissolved. The resulting solution was diluted to 5 ppm, tested in parallel 3 times, and the average value was taken.

## 4. Conclusions

The catalyst Ru/ASMA@AC was applied to the hydrogenation of glucose to sorbitol. After optimizing the reaction conditions, 1.50 g of Ru/ASMA@AC catalyst and 100 g of 20 wt.% glucose solution were reacted at 130 °C, 4.0 MPa and 600 rpm. After 3 h, the conversion of glucose was 99.69%, and the selectivity of sorbitol was 93.05%. Through the test of the reaction kinetics, it was proved that the hydrogenation of glucose was a first-order reaction, and the reaction activation energy was 73.04 kJ/mol. Furthermore, the catalytic performance of the Ru/ASMA@AC catalysts for glucose hydrogenation was compared with the traditional Ru/AC catalyst, and the Ru/ASMA@AC catalyst was not significantly inactivated after five repetitions, whereas Ru/AC catalyst suffered from a 10% decrease in sorbitol yield after three cycles. Since the Ru/ASMA@AC catalyst has shown high sorbitol yield and excellent stability in hydrogenation performance, it holds significant potential for industrial applications in the food, pharmaceutical, and cosmetic industries.

## Figures and Tables

**Table 1:** Specific surface area, pore size and pore volume of activated carbon before and after polymerization and metal coordinated.

Samples	BET Specific Surface Area (m^2^/g)	Pore Size (nm)	Pore Volume (cm^3^/g)
Pure AC	1064.83	3.44	0.60
ASMA@AC	507.99	4.47	0.35
Ru/AC	602.13	4.50	0.47
Ru/ASMA@AC	431.60	3.15	0.26

## Data Availability

Not applicable.

## Sample Availability

The samples are not available from the authors.
